# Spectroscopic and calorimetric analysis of the interaction between 9-hydroxy-5-methyl-12*(H)*-quino[3,4-*b*][1,4] benzothiazinium chloride with potential anticancer activity and main carrier plasma proteins. *In vitro* studies

**DOI:** 10.3389/fmolb.2026.1872215

**Published:** 2026-07-29

**Authors:** Aleksandra Owczarzy, Patrycja Piśla, Katarzyna Piordas, Wojciech Rogóż, Karolina Kulig, Andrzej Zięba, Małgorzata Maciążek-Jurczyk

**Affiliations:** 1 Department of Physical Pharmacy, Faculty of Pharmaceutical Sciences in Sosnowiec, Medical University of Silesia, Katowice, Poland; 2 Department of Organic Chemistry, Faculty of Pharmaceutical Sciences in Sosnowiec, Medical University of Silesia, Katowice, Poland

**Keywords:** 9-hydroxy-5-methyl-12(H)-quino[3,4-b][1,4]-benzothiazine chloride, circular dichroism spectroscopy, isothermal titration nanocalorimetry, ligand-protein interactions, main plasma carrier proteins, spectrofluorescence, UV-vis spectroscopy

## Abstract

Human serum albumin and α_1_ acid glycoprotein play a key role in the transport and distribution of many compounds in the human bloodstream. Investigating their interactions with drugs is an important part of the preliminary evaluation of the pharmacokinetic profile during the research and development of new drugs. 9-hydroxy-5-methyl-12(*H*)-quino[3,4-*b*][1,4]benzothiazinum chloride (Salt4) is a newly synthesized substance that demonstrated antiproliferative activity *in vitro* against human colon tumor and Lewis lung carcinoma cell lines. The aim of this study was to evaluate the interaction between Salt4 and HSA as well as AGP using spectroscopic and nanocalorimetric techniques and to assess the effect of Salt4 on the secondary and tertiary structure of these proteins. Spectrofluorescence spectroscopy were used to determine the Stern–Volmer constants (K_S-V_) and bimolecular quenching constants rate (k_q_). The association constants (K_a_) and the number of binding site classes (n_c_) of the complexes formed were calculated using the Klotz equation. Binding site markers dedicated to HSA and AGP were applied to identify high-affinity binding sites. Changes in the tertiary and secondary structures were evaluated using fluorescence, UV-Vis and circular dichroism spectroscopic studies. Isothermal titration nanocalorimetry (nanoITC) was used to determine thermodynamic parameters, which may suggest possible dominant contributions responsible for complex stabilization. The results indicate that Salt4 forms static and predominantly stable complexes with moderate affinity in one class of binding sites with both HSA and AGP. The Salt4-HSA and Salt4-AGP complexation processes are spontaneous and multifactorial, involving hydrophobic interactions, dehydration of the binding site, solvent reorganization, and structural adaptation of the protein; hydrophobic interactions may be the dominant type of bond responsible for the stability of the complexes under study. Salt4 affects the tertiary structure of the studied proteins but does not change their secondary structure. The preliminary results confirm that the adopted research hypothesis is promising and justify further studies.

## 1 Introduction

Human serum albumin (HSA) is the dominant protein in human blood plasma and plays a key role in maintaining the body’s internal balance. HSA concentration accounts for approximately 60% of all plasma proteins (Bteich, 2019). As a carrier protein, HSA is responsible for the transport of both exogenous and endogenous ligands, including hormones, bile acids, drugs, enzymes, metal ions, and toxins. It also helps maintain proper oncotic pressure, acid-base balance, and oxidation-reduction balance. HSA is a globular, heart-shaped plasma protein that consists of a single polypeptide chain with multiple elongated loops, composed of 585 amino acid residues. Its secondary structure is dominated mainly by the α helix structures and does not contain any β sheet elements (Mishra and Heath, 2021; Yang et al., 2014). The HSA tertiary structure is organized into three domains (I, II, III). Each of these domains is divided into two subdomains, identified as subdomains IA, IB, IIA, IIB, IIIA, and IIIB. Two key regions responsible for ligand binding have been identified in the HSA structure. They are located in subdomains IIA (Sudlow site I) and IIIA (Sudlow site II) of the HSA molecule, respectively. Sudlow site I is responsible for the transport of large, negatively charged molecules with a complex structure, such as warfarin, phenylbutazone, azapropazone, and indomethacin. The Sudlow site I has a hydrophobic character with a limited presence of polar amino acid groups, one of which is located at the bottom of the binding pocket (Tyr-150, His-242, and Arg-257), while the other are located at its entrance (Lys-195, Lys-199, Arg-218, and Arg-222). A single tryptophan residue at position 214 (Trp-214) is characteristic of this site, while the presence of numerous basic residues is a key factor determining the selectivity of this binding site. The Sudlow site II is involved in binding mainly smaller, aromatic carboxylic compounds such as ibuprofen, digitoxin, benzodiazepine, and propofol (Mishra and Heath, 2021; Alexandri et al., 2023; Owczarzy et al., 2023). This region forms a hydrophobic pocket with a single polar area located near the entrance to the binding pocket and is restricted by amino acid residues such as Tyr-411 and Arg-410. In addition to Tyr-411, two tyrosyl residues can be distinguished in the structure of Sudlow site II: Tyr-401 and Tyr-497. Sudlow site II is significantly smaller and more elongated than Sudlow site I. It also exhibits greater binding selectivity, as it is strongly dependent on the structural modification of the substance with which it interacts. Both Sudlow binding sites play a key role in the distribution of medicinal substances in the human body (Owczarzy et al., 2025a; Owczarzy et al., 2023; Yamasaki et al., 2013). In addition, recently, a third significant binding site has been described. It is located in the IB subdomain, which is allosterically coupled to Sudlow sites and considered to be the third main area of ligand binding to the HSA molecule. It is characterized by the presence of many acidic, neutral, and basic amino acid residues, which allows this subdomain to bind molecules with different chemical natures (Zsila, 2013).

α_1_ acid glycoprotein (AGP) is an acute phase glycosylating protein. The concentration of AGP accounts for approximately 1%–3% of all plasma proteins. Under physiological conditions, AGP has anti-inflammatory and immunomodulatory effects. Its concentration in plasma increases several hundredfold during cancerogenesis, inflammation, and infection. AGP also plays as a carrier protein, as it can bind and transport many endogenous molecules, like catecholamines, progesterone, free fatty acids, platelet-activating factor, and exogenous ones, including drugs like warfarin, pinometostat, aripiprazole, and imatinib (Ruiz, 2021). The AGP structure consists of a single polypeptide chain composed of 183 amino acids and a significant amount of carbohydrate structures, which account for approximately 45% of the protein’s total molecular weight and determine its negative charge. The AGP molecule is mainly composed of a β sheet structure consisting of eight anti-parallel β strands connected by loops (βA/βB, βC/βD, βE/βF, βG/βH) surrounding three α helices (α1, α2, α3). In the AGP structure, three tryptophanyl residues are identified. The first one is located at position 25 (Trp-25) and is in the deepest part of the β barrel; the next one (Trp-122) is located at the entrance to the ligand-binding pocket, which exposes it to the environment. Trp-166, on the other hand, is completely exposed on the protein surface. Due to the presence of two positively charged arginine residues (Arg-68, Arg-90) in the entrance of the binding pocket, AGP has the ability to bind negatively charged molecules (Owczarzy et al., 2023; Owczarzy et al., 2021). The main AGP binding site is located at the bottom of the β-barrel structure (Gly-23-Phe-32, Ile-44-Asn-54, Thr-59-Arg-68, Glu-71-Glu-82, Thr-87-Glu-92, Arg-95-Leu-102, Thr-109-Ser-114, and Gly-123-Ala-128). This region consists of three separate lobes (I-III). Lobe I, which is the largest and deepest cavity in the protein structure, is the main site of hydrophobic interaction with the drug. In contrast, lobes II and III are much smaller, and have a negative electrostatic character, and are part of the central lobe I. AGP exhibits the ability to bind ligands with different structures, including warfarin, prazosin, imatinib, and dipyridamole (Nishi et al., 2011).

**FIGURE 1:**
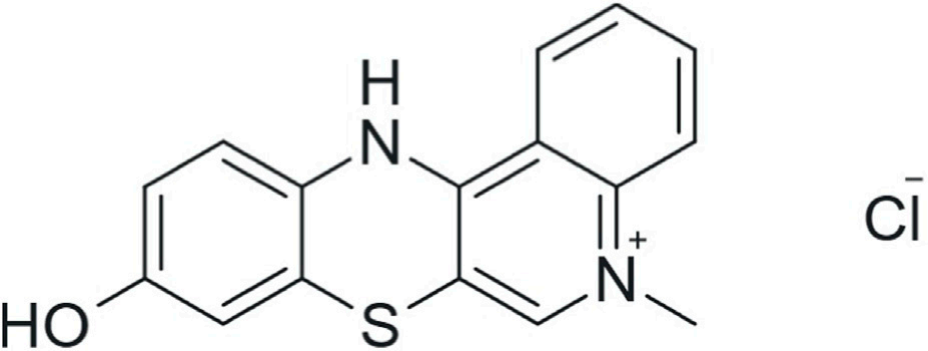
9-hydroxy-5-methyl-12*(H)*-quino [3,4-*b*][1,4]-benzothiazine chloride (Salt4) [own work].

Phenothiazines are a class of organic compounds characterized by the presence of a 10H-dibenzo [b,e]-1,4-thiazine system. Their tricyclic structure containing sulfur and nitrogen atoms predisposes them to wide use in medicine and pharmacy. Phenothiazine derivatives are subjected to structural modifications, mainly by substitution in position 10 with dialkylaminoalkyl groups and in position 2 with smaller groups, which makes this group of compounds characterized by a wide range of biological activities, including neuroleptic, antiemetic, antihistamine, antipruritic, analgesic, and antiparasitic (Posso et al., 2022; Pluta et al., 2011). Their immunomodulatory, antiviral, antibacterial, antifungal, antiproliferative, and anticancer effects have also been reported (Zięba et al., 2010). 9-hydroxy-5-methyl-12(H)-quino[3,4-b][1,4]benzothiazine chloride (Salt4) (Figure 1) is a newly synthesized derivative from the phenothiazine group. The Salt4 antiproliferative activity has been tested and confirmed *in vitro* on human colon cancer (HCT116) and Lewis lung carcinoma (LLC) cell lines in relation to doxorubicin, which is a drug commonly used in cancer chemotherapy (Zięba et al., 2010; Zięba and Bober, 2016).

Due to the numerous side effects of conventional anticancer drugs and the limited effectiveness of currently used oncological therapies, the synthesis of new substances with potential anticancer activity and the physicochemical evaluation of their interaction with major carrier proteins are an important part of the preliminary evaluation of the pharmacokinetic profile during the research and development of new drugs. Although the anticancer potential of Salt 4 has been previously described in the literature (Zięba et al., 2010; Zięba and Bober, 2016), there is currently a lack of information regarding the interaction of Salt 4 with major carrier proteins such as HSA and AGP. The lack of such data represents a significant knowledge gap, since the characteristic of protein binding is essential for an initial assessment of a new compound’s pharmacological potential and its progression to further stages of research in accordance with the ethical principles for research involving laboratory animals. The aim of the study was the first comprehensive spectroscopic and nanocalorimetric analysis of the interaction between Salt4 – a new substance with potential anticancer activity and HSA and AGP. This is the first report describing the binding of Salt4 to model carrier proteins, providing new insights into its transport characteristics in plasma and supporting the assessment of its potential as a candidate anti-cancer compound. The current work is a continuation of research on salts interactions with transport proteins.

## 2 Materials and methods

### 2.1 Materials

All reagents and compounds used were of analytical or spectroscopic grade. HSA, fraction V was purchased from MP Biomedicals (USA), while AGP was obtained from Sigma-Aldrich (Germany). The compound Salt4 was synthesized at the Department of Organic Chemistry, Faculty of Pharmaceutical Sciences in Sosnowiec, Medical University of Silesia in Katowice (Poland). Dansyl-L-phenylalanine (dPhe) was purchased from MP Biomedicals (USA). In turn, dansyl-L-glycine (dGly) and quinaldine red (QR) were obtained from Sigma-Aldrich (Germany). Spectroscopic grade methanol was supplied by Supelco (Germany). All chemicals were used without further purification.

### 2.2 Methods

According to the previously described protocols (Owczarzy et al., 2021; Owczarzy et al., 2023; Owczarzy et al., 2025a (1); Owczarzy et al., 2025b (2)), a JASCO FP-6500 spectrofluorimeter was used to register the steady-state fluorescence spectra of the following systems: Salt4-HSA ([Salt4]:[HSA] molar ratios from 0:1 to 8:1), Salt4-AGP ([Salt4]:[AGP] molar ratios from 0:1 to 8:1), Salt4-dGly-HSA ([Salt4]:[dGly]:[HSA] molar ratios from 0:1:1 to 10:1:1), Salt4-dPhe-HSA ([Salt4]:[dPhe]:[HSA] molar ratios from 0:1:1 to 10:1:1), Salt4-QR-AGP ([Salt4]:[QR]:[AGP] molar ratios from 0:1:1 to 10:1:1 and from 0:0.5:1 to 10:0.5:1). To excite the HSA and AGP fluorescence in the presence of Salt4 at increasing concentrations, the excitation wavelengths λ_ex_ 275 nm and λ_ex_ 295 nm were used. The steady-state fluorescence spectra of the Salt4-dGly-HSA and Salt4-dPhe-HSA systems were registered at the λ_ex_ 350 nm excitation wavelength. A JASCO V-760 UV-Vis spectrophotometer was used to register changes in the maximum absorption intensity of the studied systems, which allowed for the calculation of fluorescence corrected to account for the internal filter effect (IFE) caused by the absorption of radiation by the studied systems. A JASCO J-1500 CD spectropolarimeter was used to register the far-UV circular dichroism (far-UV-CD) spectra of HSA and AGP in a nitrogen atmosphere at a temperature of 25 °C. The measurements were performed in a quartz cuvette with an optical path length of 1 mm. The obtained CD spectra were corrected by subtracting the spectra obtained for the phosphate buffer and then smoothed using the Savitzky and Golay model. In turn, isothermal titration nanocalorimetry (nanoITC, TA Instrument) was used to analyze intermolecular thermodynamic interaction.

### 2.3 Theory and calculation

Due to the observed absorption of the radiation at both excitation (λ_ex_ 275 nm, λ_ex_ 295 nm) and emission wavelengths, all fluorescence values obtained for both Salt4-HSA and Salt4-AGP systems, necessary for performing the relevant calculation, were corrected for the IFE in accordance with equation Equation 1 (Kirby, 1971):

$$F_{\text{cor}}=F_{\text{obs}}\cdot \exp^{\frac{\left(A_{\text{ex}}{\cdot A}_{\text{em}}\right)}{2}}$$ (1)

where:

F_cor_, F_ob_–corrected and observed fluorescence intensity, respectively.

A_ex_, A_em_–absorption at excitation and emission wavelength, respectively.

Spectral parameter A was used to evaluate changes in the HSA and AGP tertiary structure in the presence of Salt4 based on the equation Equation 2 (Maciążek-Jurczyk et al., 2021; Turoverov et al., 1976):

$$A={\left(\frac{F_{365}}{F_{320}}\right)}_{\text{cor}}$$ (2)

where:

F_365_, F_320_ – fluorescence intensity (corrected for IFE) at λ 365 nm and λ 320 nm wavelengths, respectively.

The Stern–Volmer equation (Equation 3) was used to determine the type of HSA and AGP fluorescence quenching (static, dynamic, mixed) in the presence of Salt4 at increasing concentration (Kirby, 1971):

$$\frac{F_{0}}{F}=1+k_{q}\tau_{0}\cdot \left[L\right]=1+K_{S-V}\cdot \left[L\right]$$ (3)

where:

F_0_, F–fluorescence intensity (corrected for IFE) in the absence and presence of Salt4, respectively.

$k_{q}=\frac{K_{S-V}}{\tau_{0}}$ [mol^-1^ · L · s^-1^] – bimolecular quenching constant rate (τ_0_ – protein average fluorescence lifetime)

K_S-V_ [mol^-1^ · L] – Stern–Volmer constant

[L] – ligand (Salt4) concentration.

The Klotz equation (Equation 4) was used to determine the stability of the ligand-protein complex, and for this reason, the association constants (K_a_ [L∙mol^-1^]) and the number of binding site classes (n_c_) were calculated (Klotz and Hunston, 1971):

$$\frac{1}{r}=\frac{1}{n_{c}}+\frac{1}{n_{c}\cdot K_{a}\cdot \left[L_{f}\right]}$$ (4)

where:

r–number of ligand molecules bound to one protein molecule

n_c_–number of binding site classes.

K_a_ [mol^-1^ · L] – association constant

[L_f_] – free ligand (Salt4) concentration.

The percentage of fluorescence marker displacement, dPhe and dGly from the HSA molecule and QR from the AGP molecule, was calculated to determine the Salt4 high-affinity binding sites in the studied protein molecules based on the equation Equation 5:

$$\text{percentage of displacement}=\frac{F_{0}-F}{F_{0}}\cdot 100\%$$ (5)

where:

F_0_, F–fluorescence intensity (corrected for IFE) in the absence and presence of Salt4, respectively.

The final ellipticity, expressed as the mean residue ellipticity at wavelength λ ([θ]_MRW_) was used to determine the changes of HSA and AGP secondary structure in the presence of Salt4 based on the equation Equation 6 (Dockal et al., 2000; Kelly et al., 2005):

$${\left[\theta \right]}_{\text{mre}}=\frac{\text{MRW}\cdot \theta_{\lambda }}{10\cdot l\cdot c}\;\left[\deg\cdot {\text{cm}}^{2}\cdot {\text{dmol}}^{-1}\right]$$ (6)

where:

MRW–mean residue weight (MRW_HSA_ = 114 Da, MRW_AGP_ = 110 Da)

θ_λ_–observed ellipticity at wavelength λ [deg]

l–optical path length [cm].

### 2.4 Statistic

All measurements were performed in three independent replicates, and the results were presented as arithmetic mean ± standard deviation (SD). The obtained numerical data were processed using OriginPro software version 8.5 SR1 (Northampton, MA, USA).

## 3 Results

### 3.1 *In silico* analysis of the physicochemical, pharmacokinetic, and toxicological properties of Salt4

The preliminary analysis of the physicochemical, pharmacokinetic, and toxicological properties of Salt4 was performed *in silico* using the SwissADME and ADMETab 3.0 applications. The obtained results are summarized in Table 1.

**TABLE 1:** Predictive *in silico* analysis of the physicochemical, pharmacokinetic, and toxicological properties of 9-hydroxy-5-methyl-12(H)-quino [3,4-b][1,4]-benzothiazine chloride.

Physicochemical properties and medical chemistry	LogP_o/w_	3,188
	Water solubility	Moderate
	Numer rotatable bonds	0
	Number of H-bonds acceptors	1
	Number of H-bonds donors	2
	Lipiński rule	+
Pharmacokinetics	GI absorption	+
	Caco-2 permeability	-
	MDCK permeability	+
	PAMPA	+
	Pgp inhibitor	-
	Pgp substrate	+
	HIA	+
	PPB	+
	VDss	+
	BBB	-
	Fu	+
	BSEP inhibitor	+
Toxicity	Rat oral acute toxicity	Moderate risk after oral administration
	Human hepatotoxicity	Low risk of hepatotoxicity
	Drug-induced nephrotoxicity	Low risk of nephrotoxicity
	Ototoxicity	Very low risk of ototoxicity
	Hematotoxicity	Low risk of hematotoxicity

### 3.2 Spectroscopic analysis of Salt4-model carrier proteins complexes

**FIGURE 2:**
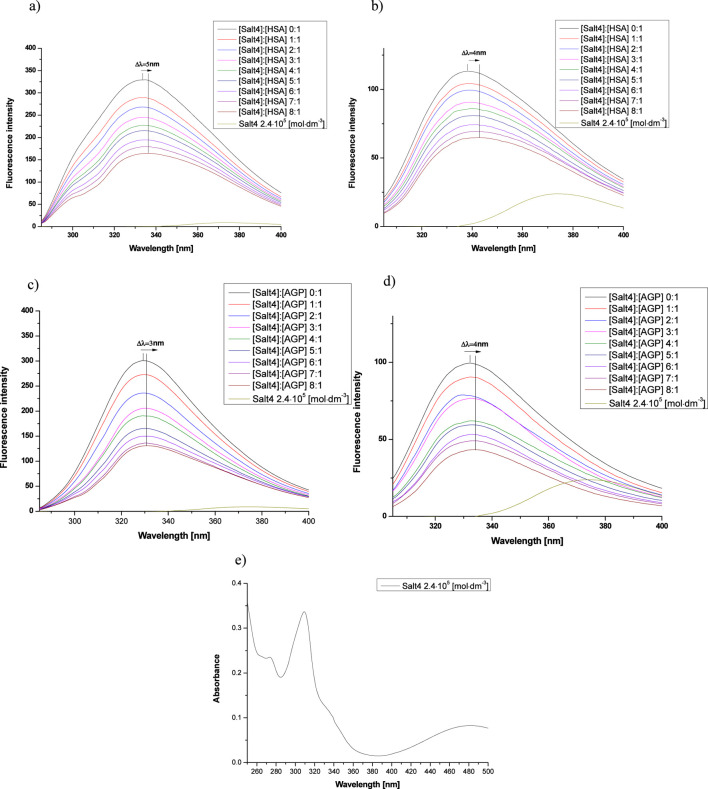
The most representative steady-state fluorescence spectra of the studied systems and the corresponding shift for the investigated Salt4-HSA (**(a)** λ_ex_ 275 nm, **(b)** λ_ex_ 295 nm) and Salt4-AGP (**(c)** λ_ex_ 275 nm, **(d)** λ_ex_ 295 nm) systems; absorption spectrum of Salt4 **(e)**.

The HSA and AGP steady-state fluorescence spectra were registered at a protein molar concentration 3 · 10^–6^ mol · L^-1^ in the presence of Salt4 at increasing concentrations, from 0 to 2.4 · 10^–5^ mol · L^-1^, and presented in Figure 2.

Based on obtained HSA and AGP steady-state fluorescence spectra, a decrease in the fluorescence intensity of both HSA and AGP in the presence of Salt4 at increasing concentrations at both excitation wavelengths λ_ex_ 275 nm (Figures 2a,c) and λ_ex_ 295 nm (Figures 2b,d) was observed. Furthermore, for both studied systems (Salt4-HSA, Salt4-AGP), a shift in the maximum fluorescence intensity towards longer wavelengths was also noticed. Due to the observed shift and the shape of the HSA and AGP steady-state fluorescence, which are broad and flattened at the maximum fluorescence intensity, based on Equation 2, spectral parameter A was calculated. The obtained data are summarized in Table 2.

**TABLE 2:** Spectral parameter A for HSA and AGP in the absence and presence of Salt4 at increasing concentration from 0 to 2.4 10^−5^ mol L^−1^ (λ_ex_ 275 nm and λ_ex_ 295 nm).

Model carrier protein	Salt4 [mol·L^−1^]	λ_ex_ 275 nm		λ_ex_ 295 nm	
		λ_max_±SD^*^ [nm]	Parameter A±SD^*^	λ_max_±SD^*^ [nm]	Parameter A±SD^*^
**HSA**	0	334.5 ± 0.7	0.75 ± 0.0	338.5 ± 0.7	1.12 ± 0.0
	2.4 10^−5^	336.0 ± 1.4	1.00 ± 0.1	342.5 ± 0.7	1.34 ± 0.3
**AGP**	0	329.0 ± 0.6	0.51 ± 0.1	331.0 ± 1.4	0.70 ± 0.1
	2.4 10^−5^	332.0 ± 0.0	0.72 ± 0.0	336.0 ± 0.0	1.07 ± 0.1

^*^ SD, standard deviation.

Based on the data collected in Table 2, due to the presence of Salt4 at increasing concentrations, both an increase in the wavelength at which the maximum fluorescence intensity occurs and the spectral parameter A values were observed (λ_ex_ 275 nm and λ_ex_ 295 nm).

**FIGURE 3:**
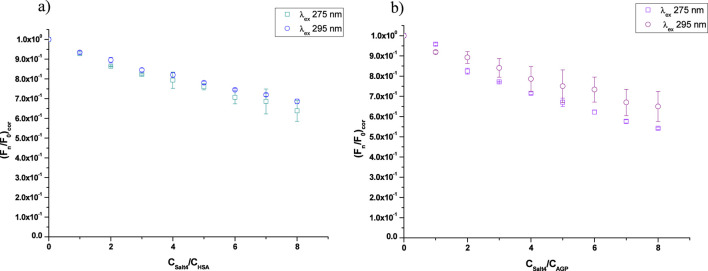
Fluorescence quenching curves of **(a)** HSA and **(b)** AGP in the presence of Salt4 at increasing concentration from 0 to 2.4 10^−5^ mol L^−1^ (λ_ex_ 275 nm and λ_ex_ 295 nm).

Based on the HSA and AGP steady-state fluorescence spectra in the presence of Salt4 at increasing concentrations, the fluorescence quenching curves were plotted (λ_ex_ 275 nm and λ_ex_ 295 nm, Figure 3).

The HSA and AGP fluorescence quenching was observed in the presence of Salt4 at increasing concentrations at both λ_ex_ 275 nm and λ_ex_ 295 nm excitation wavelengths. Additionally, the Salt4-HSA system quenching curves at the λ_ex_ 275 nm excitation wavelength overlap with the curve obtained at λ_ex_ 295 nm. In contrast, the Salt4-AGP system quenching curves excited at λ_ex_ 275 nm and λ_ex_ 295 nm wavelengths have a different course. Moreover, based on the register steady-state fluorescence spectra of the studied systems (Figure 2) and the obtained fluorescence quenching curves (Figure 3), the percentage of HSA and AGP fluorescence quenching in the presence of Salt4 at increasing concentration was calculated (Equation 3) and summarized in Table 3.

**TABLE 3:** The percentage of HSA and AGP fluorescence quenching in the presence of Salt4 at increasing concentration from 0 to 2.4 · 10^−5^ mol L^−1^ (λ_ex_ 275 nm and λ_ex_ 295 nm).

[Salt4]: [model carrier protein] molar ratio	Fluorescence quenching [%]±SD^*^	
	λ_ex_ 275 nm	λ_ex_ 295 nm
[Salt4]:[HSA] 8:1	36.88 ± 3.87	31.32 ± 1.18
[Salt4]:[AGP] 8:1	45.80 ± 0.58	53.80 ± 3.84

^*^ SD, standard deviation.

**FIGURE 4:**
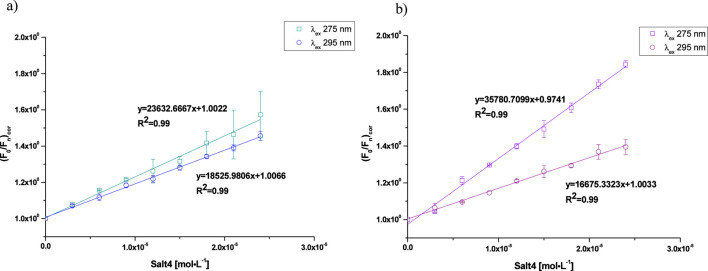
The Stern–Volmer curves for **(a)** HSA and **(b)** AGP in the presence of Salt4 at increasing concentration from 0 to 2.4 · 10^−5^ mol L^−1^ (λ_ex_ 275 nm and λ_ex_ 295 nm).

Based on the data collected in Table 3, it can be observed that the presence of Salt4 quenches AGP fluorescence more strongly than HSA, by approximately 9% (λ_ex_ 275 nm) and 15% (λ_ex_ 295 nm). According to the Stern–Volmer Equation 3, the Stern–Volmer curves have been plotted for both model carrier proteins in the presence of Salt4 at increasing concentrations (λ_ex_ 275 nm and λ_ex_ 295 nm). The obtained curves have been shown in Figure 4.

For the studied Salt4-HSA and Salt4-AGP systems, the Stern–Volmer constants (K_S-V_ [L·mol^-1^]) and the bimolecular quenching constants rate (k_q_ [L·mol^-1^·s^-1^]) were calculated. The obtained results are presented in Table 4.

**TABLE 4:** The Stern–Volmer constants (K_S-V_) and bimolecular quenching constants rate (k_q_) (λ_ex_ 275 nm and λ_ex_ 295 nm).

(Salt4-model carrier protein)_system_	λ_ex_ 275 nm		λ_ex_ 295 nm	
	(K_s-v_±SD*) · 10^4^ [mol^-1^·L]	(k_q_±SD*) · 10^12^ [mol^-1^·L·s^-1^]	(K_s-v_±SD*) · 10^4^ [mol^-1^·L]	(k_q_±SD*) · 10^12^ [mol^-1^·L·s^-1^]
(Salt4-HSA)_system_	2.23 ± 0.37	3.94 ± 0.63	1.85 ± 0.05	3.08 ± 0.12
(Salt4-AGP)_system_	3.58 ± 0.05	5.92 ± 0.07	2.25 ± 0.91	3.75 ± 1.52

^*^ SD, standard deviation.

The K_S-V_ and the k_q_ obtained for the Salt4-HSA and Salt4-AGP systems were calculated to be in the order of magnitude 10^4^ L mol^-1^ and 10^12^ L mol^-1^·s^-1^, respectively. Moreover, based on the presented data, it was observed that the values of the K_S-V_ and k_q_ are higher for the Salt4-AGP system at λ_ex_ 275 nm than at λ_ex_ 295 nm excitation wavelengths.

**FIGURE 5:**
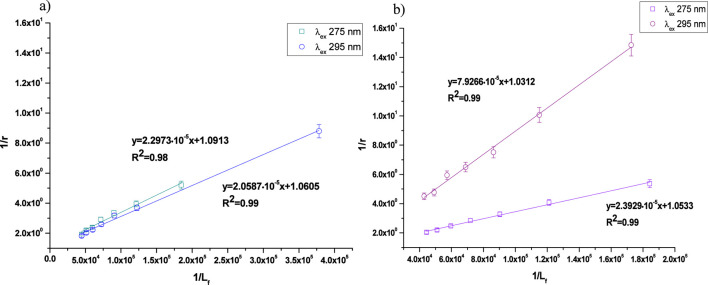
The Klotz curves for the **(a)** Salt4-HSA and **(b)** Salt4-AGP systems (λ_ex_ 275 nm and λ_ex_ 295 nm).

Based on Klotz Equation 4, Klotz curves were plotted for the Salt4-HSA and Salt4-AGP systems (λ_ex_ 275 nm and λ_ex_ 295 nm, Figure 5). The association constants of the studied systems (K_a_ [L·mol^-1^]) and the number of binding site classes (n_c_) were calculated and presented in Table 5.

**TABLE 5:** Binding parameters for Salt4-model carrier protein systems (λ_ex_ 275 nm and λ_ex_ 295 nm).

(Salt4-model carrier protein)_system_	λ_ex_ 275 nm		λ_ex_ 295 nm	
	(K_a_ *±*SD*) · 10^4^ [mol^−1^ L]	n±SD*	(K_a_ *±*SD*) · 10^4^ [mol^−1^ L]	n±SD*
**(Salt4-HSA)**_**system**_	4.75 ± 1.06	0.93 ± 0.05	4.68 ± 0.99	0.95 ± 0.03
**(Salt4-AGP)**_**system**_	4.38 ± 1.27	0.95 ± 0.01	4.40 ± 0.50	0.97 ± 0.04

^*^ SD, standard deviation.

Based on the data presented in Table 5, it was observed that the K_a_ of both studied systems (λ_ex_ 275 nm and λ_ex_ 295 nm) are in the order of magnitude 10^4^ [L·mol^-1^], and the n_c_ values oscillates around 1.

To evaluate the high-affinity binding sites of Salt4 in the HSA and AGP molecules, the steady-state fluorescence spectra of dGly and dPhe as well as QR were registered. Then, based on Equation 5, the percentage of fluorescent marker displacement from binding sites in HSA (Table 6) and AGP (Table 7) molecules was calculated.

**TABLE 6:** The percentage of fluorescent marker displacement (dGlu and dPhe) from HSA binding sites.

C_Salt4_ [mol·L^-1^]	[HSA]: [dGly]	[HSA]: [dPhe]
	1:1	
	Percentage of displacement [%]±SD*	
0	—	—
3·10^−5^	39.24 ± 0.41	49.41 ± 3.14

^*^ SD, standard deviation.

**TABLE 7:** The percentage of QR displacement from AGP binding sites.

C_Salt4_ [mol·L^-1^]	[AGP]: [QR]	
	1:0.5	1:1
	Percentage of displacement [%]±SD*	
0	—	—
3·10^−5^	62.64 ± 1.19	65.89 ± 1.18

^*^ SD, standard deviation.

Based on the data collected in Table 6, it was observed that the presence of Salt4 at a concentration from 0 to 2.4 · 10^−5^ mol L^-1^ causes the displacement of fluorescent markers from the HSA molecule. A higher percentage of displacement was observed for dPhe (49.41% ± 3.14%) than for dGly (39.24% ± 0.41%).

Based on the data collected in Table 7, it can be observed that the differences in the percentage of QR displacement from the binding site in the AGP molecule are not significant for both molar ratios and fluctuate around 65%.

**FIGURE 6:**
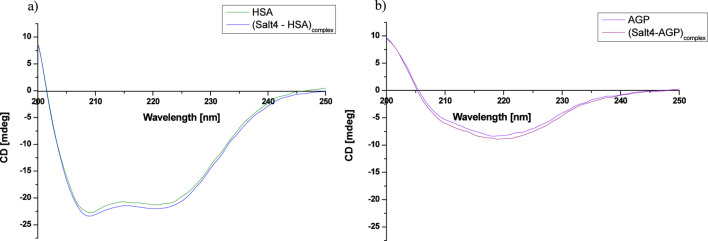
far-UV-CD spectra for **(a)** HSA and **(b)** AGP, both in the absence and presence of Salt4 at concentration 2.4 10^−5^ mol L^-1^.

To assess the Salt4 effect on the HSA and AGP secondary structure, far-UV-CD spectra have been registered, both for HSA and AGP in the absence and in the presence of Salt4, and presented in Figures 6a,b, respectively. Using the BeStSel application and Reed’s reference model, the percentage contribution of secondary structure individual elements (α helix, β sheets, turn, and random) of the studied model carrier proteins (HSA, AGP) was determined. In addition, using Equation 6, the mean residue ellipticity [Θ_MRW_] of HSA and AGP, both in the absence and presence of Salt4, was determined. All obtained data are summarized in Table 8.

**TABLE 8:** Mean residue elasticity [Θ_MRW_] and the percentage content of HSA and AGP secondary structure in the absence and presence of Salt4 (BeStSel and Reed’s reference model, respectively).

	[θ]_MRW_±SD* λ_209 nm_ [deg·cm^2^·dmol^-1^]	[θ]_MRW_±SD* λ_220.4__nm_ [deg·cm^2^·dmol^-1^]	[θ]_MRW_±SD* λ_219.6__nm_ [deg·cm^2^·dmol^-1^]	%±SD* α-helix	%±SD* β-sheet	%±SD* other
HSA^a^	−19700.7 ± 383.53	−18468.5 ± 357.07	—	52.30 ± 2.26	—	47.70 ± 2.69
(Salt4-HSA)_system_ ^a^	−18468.5 ± 375.07	−18819.1 ± 49.56	—	54.55 ± 2.40	—	45.45 ± 2.90
AGP^b^	—	—	−8881.82 ± 424.32	15.45 ± 2.62	82.65 ± 0.92	—
(Salt4-AGP)_system_ ^a^	—	—	−9730.21 ± 156.33	21.20 ± 1.00	78.80 ± 1.00	—

* SD, standard deviation.

^a^ BeStSel application.

^b^ Reeds’a reference model.

Analyzing the far-UV CD spectra of studied systems, it was observed that the presence of Salt4 slightly changes the HSA and AGP far-UV-CD spectra.

Based on the data collected in Table 8, it was observed that the presence of Salt4 slightly affects the secondary structure of HSA and AGP. However, more significant changes were observed for AGP than for HSA.

### 3.3 Nanocalorimetric analysis of Salt4-model carrier proteins systems

**FIGURE 7:**
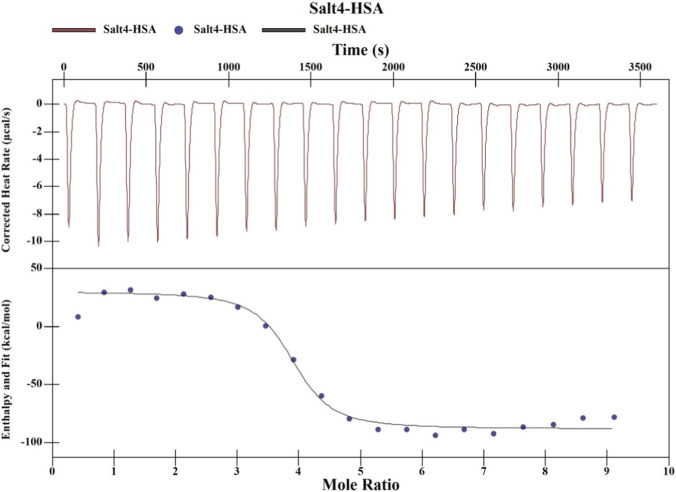
nanoITC thermogram for Salt4-HSA complex formation. The upper part of the figure shows the dependence of energy flow in the form of heat transfer obtained as a result of successive injections on time [s]. The lower part of the figure shows the binding isotherm determined by plotting the heat peak areas in the molar ratio [Salt4]:[HSA] 0.4202:1÷9.109:1. The lines show the optimized fit of the models used. Error bars do not exceed the obtained points (T = 298 K).

**FIGURE 8:**
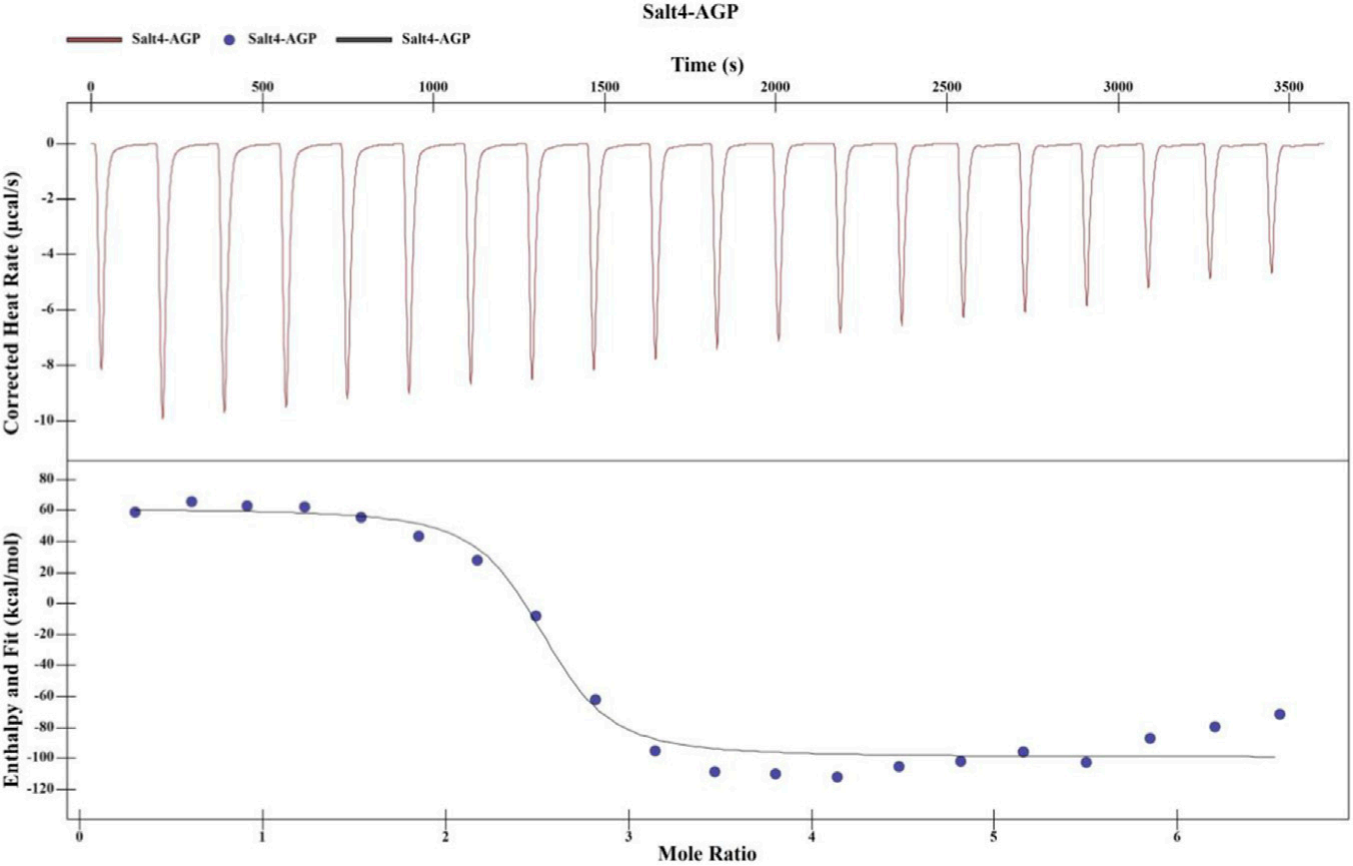
nanoITC thermogram for Salt4-AGP complex formation. The upper part of the figure shows the dependence of energy flow in the form of heat transfer obtained as a result of successive injections on time [s]. The lower part of the figure shows the binding isotherm determined by plotting the heat peak areas in the molar ratio [Salt4]: [AGP] 0.3025:1÷6.5590:1. The lines show the optimized fit of the models used. Error bars do not exceed the obtained points (T = 298 K).

Using the nanoITC, thermograms and binding isotherms of HSA and AGP in the presence of Salt4 were determined (Figures 7, 8).

Based on the obtained nanoITC thermograms (Figures 7, 8), it was observed that the binding isotherms of Salt4, both in the presence of HSA and AGP, have a clearly reversed sigmoidal course, typical of thermodynamically controlled ligand-protein interaction. They exhibit a regular shape with one distinct inflection point. In addition, based on the obtained data, the binding parameters, such as K_a_ [L·mol^-1^], stoichiometry reaction (n), and thermodynamic parameters (enthalpy change (ΔH [kcal·mol^-1^]), entropy change (ΔS [cal·mol^-1^·K^−1^]) as well as values of Gibbs free energy change (ΔG [kcal·mol^-1^]) of the studied systems, were calculated. The results are summarized in Table 9.

**TABLE 9:** Binding and thermodynamic parameters of Salt4-HSA and Salt4-AGP complexes formed.

		K_a_±SD [L·mol^-1^]	n^**^±SD	ΔH [kcal·mol^-1^]	ΔS [cal·mol^-1^·K^−1^]	ΔG [kcal·mol^-1^]
Salt4	HSA	(4.74 ± 1.15)·10^6^	3.71 ± 0.01	107.60 ± 15.28	381.35 ± 64.8	−6.05 ± 4.04
	AGP	(2.10 ± 0.68)·10^6^	2.38 ± 0.16	136.77 ± 0.35	492.10 ± 0.12	−9.97 ± 0.19

* SD, standard deviation.

** n–stoichiometry reaction.

The obtained K_a_ values are in the order of magnitude10^6^ [L·mol^-1^] for both Salt4-HSA and Salt4-AGP systems. The n parameter values for the Salt4-HSA system fluctuate around 4, while for the Salt4-AGP system, it fluctuates around 2. Additionally, based on the data presented in Table 9, it can be observed that the reaction of Salt4 complex formation with the main carrier proteins results in positive values of both ΔH [kcal·mol^-1^] and ΔS [cal·mol^-1^·K^−1^] as well as negative values of ΔG [kcal·mol^-1^].

## 4 Discussion

### 4.1 *In silico* analysis of the physicochemical, pharmacokinetic, and toxicological properties of Salt4

An *in silico* analysis conducted using the SwissADME (Daina et al., 2017) and ADMETlab 3.0 (Fu et al., 2024) applications was shown a favorable physicochemical profile of Salt4 (Table 1), suggesting its potential as a drug candidate in future. Furthermore, the analysis showed that Salt4 exhibits moderate solubility in water and lipophilicity, which contributes to its ability to penetrate biological membranes (LogP = 3.188), enhances its ability to penetrate biological membranes. Salt4 also has one hydrogen-bond acceptor and two hydrogen-bond donors, and its molecular weight is less than 500 Da, which means that the studied substance complies with Lipinski rule. Moreover, the absence of rotatable bonds may indicate that Salt4 possesses high structural rigidity, which could lead to increased metabolic stability and interaction selectivity with the molecular target (Lipiński et al., 2001). The obtained pharmacokinetic parameters may indicate a favorable absorption profile. Positive results for human intestinal absorption (HIA) and gastrointestinal (GI) absorption may indicate effective absorption after oral administration (Di and Kerns, 2016). Additionally, the results of the PAMPA and MDCK models indicate that the studied substance exhibit good passive diffusion capacity through biological membranes. Simultaneously, the negative results of Caco-2 model may indicate the existence of limitation regarding transport through the intestinal epithelium or the active involvement of active transport mechanisms (Williams et al., 2022). The *in silico* analysis also showed that Salt4 could be a substrate of P-glycoprotein (P-gp), however, it did not exhibit any inhibitory effect against this transporter. Despite the low risk of drug interactions associated with P-gp inhibition, this result may indicate the possibility of a partial reduction in bioavailability (Giacomini et al., 2010). In turn, the analysis of selected distribution parameters reveled the possibility of plasma protein binding (PPB) and volume of distribution at steady-state (VDss) (Fu et al., 2024). Additionally, *in silico* analysis indicates the presence of a promising reservoir of free fraction (pharmacologically active) of the studied substances (Di and Kerns, 2016). In turn, toxicological assessments indicated a moderate risk after oral administration in rats, as well as low risk of hepatotoxicity, nephrotoxicity, and hematotoxicity, and a very low risk of ototoxicity. This may suggest a favorable safety profile (Dawson et al., 2012). In summary, Salt4 exhibits a promising physicochemical, pharmacokinetic, and toxicological profile *in silico*, consistent with the requirements for oral drug candidates. Consequently, spectroscopic and calorimetric analyses of the interaction between Salt4 and major model carrier protein have been conducted.

### 4.2 Spectroscopic analysis of Salt4-model carrier proteins systems

HSA and AGP fluorescence are determined by the presence of aromatic amino acid residues, mainly tyrosyl and tryptophanyl, located at high-affinity binding sites. The most important aromatic amino acid residues presented in HSA molecules include Trp-214 (subdomain IIA) and Tyr-401, Tyr-411, and Tyr-497 (subdomain IIIA) (Sudlow et al., 1972). In turn, those presented in AGP molecules are Trp-25, situated within the β-barrel, Trp-122, located at the drug-binding pocket entrance and slightly exposed to the aqueous environment, and Trp-166, located on the surface of the AGP molecule and exposed to the external environment. AGP contains several tyrosyl residues, including Tyr-27, Tyr-47, Tyr-56, and Tyr-91, which contribute to their intrinsic fluorescence and may participate in ligand–protein interaction (Zsila et al., 2020). At excitation wavelength λ_ex_ 275 nm, both tyrosyl and tryptophanyl residues are excited to fluorescence, while at excitation wavelength λ_ex_ 295 nm, only tryptophanyl residue(s). Fluorescence quenching occurs when the distance between the quencher (ligand) and protein is no greater than 10 nm (Van de Weert and Stella, 2011). Analysis of the steady-state fluorescence (Figure 2) and absorption intensities (data not shown) of HSA and AGP in the presence of Salt4 at increasing concentrations allowed the characterization of intermolecular interaction. Based on the recorded steady-state fluorescence spectra of HSA (Figures 2a,b) and AGP (Figures 2c,d), a decrease in the protein fluorescence intensity at both excitation wavelengths (λ_ex_ 275 nm, λ_ex_ 295 nm) was observed. This phenomenon was associated with the energy transfer from excited protein aromatic amino acid residues to ligand (Maciążek-Jurczyk et al., 2012). The maximum intensity of steady-state fluorescence spectra, their shape, and shift correlate closely with the type of protein and depend on experimental conditions and external factors. Their changes may indicate the interaction between the studied ligand and the carrier protein (Van de Weert and Stella, 2011). Broad and flattened steady-state fluorescence spectra of HSA and AGP in the presence of Salt4 at increasing concentrations have been registered. Although accurate determination of maximum fluorescence intensity (λ_max_) position was difficult, a shift in the maximum fluorescence intensity of proteins (HSA and AGP) towards long wavelengths (red shifts) was observed at both λ_ex_ 275 nm (Δλ_HSA_ 5 nm and Δλ_AGP_ 3 nm; Figures 2a,c) and λ_ex_ 295 nm (Δλ_HSA_ 4 nm and Δλ_AGP_ 4 nm; Figures 2b,d) excitation wavelengths. To confirm the exact values of these parameters and prove the influence of certain factors on the protein structure, due to sensitivity to slight changes in the position of the maximum fluorescence wavelength (λ_max_), spectral parameter A (Equation 2) was calculated (Parkhomenko et al., 2002; Turoverov et al., 1976). The spectral parameter A is defined as the ratio of fluorescence intensities at wavelengths of 320 and 365 nm and is highly sensitive to changes in the shape and position of the emission fluorescence spectra. Analysis of its changes (decrease or increase) provides information about conformational rearrangements affecting the tertiary structure of proteins. Wavelengths λ 320 and λ 365 nm are located on the opposite sides of a typical protein emission fluorescence spectrum. A blue shift in the fluorescence spectrum is associated with a more hydrophobic and structurally compact environment around the tryptophan residues, causes a decrease in fluorescence intensity at 365 nm relative to 320 nm, leading to lower values of the spectral parameter A. Conversely, a red shift resulting from increased exposure of aromatic amino acid residues to the solvent increases the value of the spectral parameter A. Therefore, spectral parameter A gives a sensitive indicator of spectral shifts and conformational changes. For both HSA and AGP, an increase in the spectral parameters A values were observed (Table 2). Owczarzy et al. (2021) analyzed the interaction between quinobenzothiazine derivative (Salt1) and major blood serum proteins, and similar to the present study, they registered a decrease in the fluorescence intensity of HSA and AGP in the presence of ligand at increasing concentration. Furthermore, the authors calculated the spectral parameter A and also observed an increase in this value. This phenomenon confirms the shift of the maximum fluorescence intensity of proteins towards long wavelengths. The analyzed changes were attributed to the changes in the protein tertiary structure in the presence of a ligand at increasing concentration and an increase in the hydrophilicity of the aromatic amino acid residues environment (Owczarzy et al., 2021). Moreover, Owczarzy et al. (2025a) also analyzed the values of spectral parameter A in the study on the analysis of human blood serum albumin and albumin nanoparticles as carriers of 10-(2′-pyrimidyl)-3,6-diazaphenothiazine. In turn, in this study, the authors noted a hypsochromic shift (towards short wavelengths–blue shift) in the maximum fluorescence intensity (λ_ex_ 275 nm and 295 nm), and contrary to the data collected in Table 2, a decrease in the spectral parameter A values were noted. They attributed this phenomenon to the modification of HSA tertiary structure caused by the presence of the ligand and the changes in the environment around the tryptophanyl and tyrosyl residues to more hydrophobic (Owczarzy et al., 2025b). Resuming, based on the recorded steady-state fluorescence spectra (Figure 2), the data collected in Table 2, and the cited literature, it can be concluded that Salt4 interacts with HSA and AGP. In addition, Salt4 may affect protein tertiary structure and cause an increase in the hydrophilicity of the environment of aromatic amino acid residues located in the studied proteins.

The excitation wavelength λ_ex_ 275 nm excites to fluorescence both tyrosyl and tryptophanyl residues, while the wavelength λ_ex_ 295 nm excites only the tryptophanyl residues (Owczarzy et al., 2025c). Based on Figure 3a, it was observed that HSA fluorescence quenching curves in the presence of Salt4 at increasing concentrations from 0 to 2.4 10^−5^ mol L^-1^, at both excitation wavelengths, exhibited a similar pattern and overlapped each other (Figure 3a). In turn, the AGP fluorescence quenching curves in the presence of Salt4 did not overlap (Figure 3b). In an *in vitro* study conducted by Szkudlarek et al. (Szkudlarek et al., 2020) concerning the acetohexamide binding to glycated blood serum albumin in the absence and presence of fatty acids, at excitation wavelengths λ_ex_ 275 nm and λ_ex_ 295 nm, an almost identical HSA fluorescence quenching curve was obtained in the system with acetohexamide and in the absence of fatty acids. However, the authors noted a significant difference in the course of the HSA fluorescence quenching curve in the presence of both acetohexamide and fatty acids (λ_ex_ 275 nm and λ_ex_ 295 nm). Based on these findings, they concluded that in the absence of fatty acids, tryptophanyl residue (Trp-214), located in subdomain IIA, plays a crucial role in the interaction between acetohexamide and glycated HSA, whereas in the presence of fatty acids, in the interaction between the glycated protein and the drug, both tryptophanyl and tyrosyl residues take part (Szkudlarek et al., 2020). Therefore, it can be assumed that the tryptophanyl residue (Trp-214) present in the HSA structure plays a key role in the interaction between Salt4 and HSA, while the contribution of tyrosyl residues appears to be negligible. In contrast, in the interaction between Salt4 and AGP, both tyrosyl and tryptophanyl residues seem to participate. The data collected in Table 3 indicate that in the presence of Salt4, at the [Salt4]:[AGP] 8:1 M ratio, AGP fluorescence quenching amounted to 46% and 47% at both λ_ex_ 275 nm and λ_ex_ 295 nm excitation wavelengths, respectively. In turn, slightly lower percentages of fluorescence quenching were calculated for the Salt4-HSA system, and equal to 37% (λ_ex_ 275 nm) and 31% (λ_ex_ 295 nm). The obtained results might indicate that Salt4 interacts more strongly with AGP than with HSA. For comparison, when Owczarzy et al.(2021) focused on the analysis of the interaction between quinobenzothiazine derivative and selected serum proteins, stronger fluorescence quenching of AGP than of HSA in the presence of Salt1 was observed, and therefore they concluded that this ligand exhibits higher affinity to AGP.

Analysis of protein fluorescence quenching in the presence of ligand at increasing concentrations is an important method for assessing ligand affinity to protein. This phenomenon is associated with the deactivation of excited fluorophores in the presence of the quencher (ligand) (Lakowicz, 2006). The mechanism of fluorescence quenching can be static, dynamic, or mixed (static-dynamic). Static quenching occurs when a non-fluorescent complex is formed in the ground state between the donor and acceptor. Dynamic quenching occurs as a result of molecular collisions, causing the excited fluorophores to return to their ground state. Both mechanisms lead to a decrease in the total measured fluorescence intensity of the protein (Yamasaki et al., 2013; Lichota et al., 2022).

To determine the mechanism of HSA and AGP fluorescence quenching in the presence of Salt4 at increasing concentrations, the Stern–Volmer Equation 3 was used, and Stern–Volmer curves were plotted (Figure 4). For the studied Salt4-HSA and Salt4-AGP systems the curves were linear, both at λ_ex_ 275 nm and λ_ex_ 295 nm excitation wavelengths. It is worth noting that due to the linear course of the Stern–Volmer curves, it is not possible to clearly determine the nature of protein fluorescence quenching. Therefore, the K_S-V_ and k_q_ for Salt4-HSA and Salt4-AGP were calculated (Table 4). According to Lakowicz’s theory (Lakowicz, 2006), when the bimolecular fluorescence quenching constant rate equals 10^10^ [L· mol^-1^·s^-1^], the dynamic nature of fluorescence quenching takes place. In the present study, k_q_ values of 10^12^ [L· mol^-1^·s^-1^] were obtained, and it was significantly higher than the maximum diffusion-controlled quenching rate constant reported for biomolecular systems in aqueous solution reported by Lakowicz (2006). Based on this, it can be concluded that the mechanism of fluorescence quenching of both studied systems (Salt4-HSA, Salt4-AGP), regardless of the excitation wavelengths, has a predominantly static nature. In turn, the K_S-V_ describes the distance between the ligand and the excited fluorophore. The higher the K_S-V_ value, the closer the ligand is to the fluorophore, and the stronger complex is formed. In the present study, K_S-V_ values of 10^4^ [L·mol^-1^] were obtained. Owczarzy et al. (2023) in the study concerning the spectroscopic analysis of the interaction between Salt2 and major carrier proteins calculated K_S-V_ and k_q_ values to determine the mechanism of fluorescence quenching. Similarly to the present study, they also obtained K_S-V_ and k_q_ values of 10^4^ [L·mol^-1^] and 10^12^ [L· mol^-1^·s^-1^], respectively, and indicated that Salt2 forms weak static complexes with the major carrier plasma proteins. Taking into account the results conducted by Owczarzy et al. (2023), it can be assumed that Salt4 forms stable, static complexes with both HSA and AGP (Lichota et al., 2022).

The K_a_ [L·mol^-1^] characterizes the stability of the complexes formed. Additionally, the complexity and diversity of the sites at which the ligand can interact with the protein are determined by the n_c_ (Lissi and Abuin, 2011). To determine K_a_ [L·mol^-1^] and the n_c_ for the Salt4-HSA and Salt4-AGP systems, the Klotz Equation 4 was applied. Based on the Klotz curves (Figure 5) and the calculated binding parameters (Table 5), the K_a_ values obtained for both Salt4-HSA and Salt4-AGP complexes were on the order of magnitude 10^4^ [L·mol^-1^] (at both λ_ex_ 275 nm and λ_ex_ 295 nm excitation wavelengths). However, slightly higher K_a_ values were obtained for the Salt4-HSA system. The n_c_ values oscillates within the range of 1. In the studies conducted by Vukic et al. (2020), involving the analysis of the binding capacity of natural naphthoquinone derivatives to HSA, the authors, using the Klotz method, and obtained significantly higher association constants than in the presented study, in the order of magnitude 10^7^, and assessed the affinity for all studied complexes as strong. In turn, Mariño-Ocampo et al. (2023) used spectroscopic techniques to investigate the interaction between factor Xa inhibitors (FXa) and human serum albumin and reported association constants on the order of 10^4^. Based on these results, they concluded that HSA exhibits moderate affinity to FXa inhibitors. In accordance with the literature data, it can be assumed that Salt4 forms moderately stable, static complexes with HSA and AGP in one class of binding sites of these model carrier proteins.

Dansylated amino acids (DNSA) are commonly used to identify high-affinity binding sites in proteins. In the present study, two fluorescent markers specific to the HSA molecule (dGly and dPhe) were used to predict the HSA binding site for Salt4. dGly and dPhe are recognized as fluorescent markers for Sudlow sites I and II, respectively (Sudlow et al., 1972). Based on the steady-state fluorescence spectra of the DNSA (data not shown), the percentage of fluorescence marker displacement from their binding sites in the HSA molecule by the presence of Salt4 at increasing concentrations has been calculated (Equation 4; Table 6). Based on the obtained data, it was observed that the presence of Salt4 at increasing concentrations led to the displacement of both fluorescent markers from the HSA molecule. However, the displacement percentage was higher for dPhe than for dGly. These findings suggest that Salt4 preferentially binds to/or interacts with Sudlow site II (subdomain IIIA), although interactions with Sudlow site I (subdomain IIA) cannot be excluded. In order to characterize the main binding sites in the AGP molecule, QR was used. QR is one of the most commonly applied fluorescent markers for investigating binding sites in AGP molecules with clinical relevance (Imamura et al., 1993; Marciniak et al., 2024). Using Equation 5, based on the obtained steady-state fluorescence spectra (data not shown), the percentage of QR displacement from the AGP molecule in the presence of Salt4 at increasing concentrations was calculated (Table 7). For the molar ratios [AGP]: [QR] 1:0.5 and 1:1, the differences in the percentage of QR displacement are negligible, with values of approximately 65% observed in both cases. In a study conducted by Marciniak et al. (2024) involving the interaction of isoquinoline derivatives with plasma proteins, the authors also used QR to analyze the main binding sites of ligands in the AGP molecule. Similarly to the present study, an increase in the percentage of fluorescent marker displacement was observed with increasing concentrations of isoquinoline derivatives, indicating the replacement of QR in the binding pocket by the studied alkaloids. Based on the performed experiment, it can be assumed that the binding site of Salt4 in AGP overlaps with the QR binding site, which is the main binding site of Salt4 with significant clinical relevance.

Analysis of the far-UV-CD spectra allows for the evaluation of the secondary structure changes of the studied proteins. However, combining CD spectroscopy with appropriate analysis using the BeStSel application and Reeds’ reference model provides precise information on the percentage content of individual secondary structural elements (α-helices and β-sheets) and their possible conformational changes as a result of interaction with the ligand (Kardos et al., 2025; Micsonai et al., 2022; Chudzik et al., 2016). As shown in the registered far UV CD spectra of the studied proteins in the absence and presence of the ligand (Figure 6), the HSA far-UV-CD spectrum has two characteristic negative bands at wavelengths λ 209 nm and λ 220.4 nm (Figure 6a), which confirms its dominant α helical structure (Rogóż et al., 2022). In turn, the only one negative band at approximately 222 nm in the AGP far-UV-CD spectrum (Figure 6b) indicates the predominant β structure of this protein (Chudzik et al., 2016). The presence of Salt4 resulted in slight alterations in the CD spectra of both proteins. Therefore, it can be assumed that the presence of the ligand may slightly influence the HSA and AGP secondary structure. The mean residual ellipticity values were calculated using Equation 5. Furthermore, the percentage content of HSA and AGP secondary structure in the absence and presence of Salt4 was determined using the BeStSel application and Reed’s reference model, respectively (Table 8).

As shown in Table 8, the percentage content of HSA and AGP secondary structure elements does not change significantly as a result of the presence of Salt4. The largest difference was observed for AGP, where the percentage of α helix content increased by approximately 5% in the presence of the ligand. Munro et al. (1994) demonstrated that the binding of the main BM3 domains to cytochrome P-450 is not accompanied by a change in its secondary structure. However, the authors of the cited study reported that the domain interactions were associated with local structural rearrangements, reflected by alterations in the environment of amino acids and the heme group. Moreover, changes in the CD spectral profile and mean residue ellipticity values provided evidence for complex formation (Munro et al., 1994). Based on the obtained results, it can be concluded that the presence of Salt4 does not induce conformational changes in the secondary structures of the main carrier proteins. Consequently, it does not interfere with their biological function, which may have important clinical implications. Furthermore, the slight changes observed provide further evidence for the Salt4-HSA and Salt4-AGP complex formation.

### 4.3 Nanocalorimetric analysis of Salt4-model carrier proteins complexes

To characterize the type of interaction occurring between Salt4 and HSA and AGP, nanoITC was used. This technique allows for the general analysis of the thermodynamic profile of the studied systems and the determination of the predominant type of bonds involved in the ligand-protein complex formation (Rogóż et al., 2022; Owczarzy et al., 2025c). nanoITC thermograms for Salt4-HSA (Figure 7) and Salt4-AGP (Figure 8) systems confirmed the energy transfer occurring during complex formation, while based on the HSA and AGP binding isotherms with Salt4 (Figures 7, 8, respectively), the sigmoidal course of isotherms with one distinct inflection point has been registered. Subsequently, the obtained thermograms and isotherms were used to determine the general thermodynamic profile and binding parameters (Table 9). K_a_ [L·mol^-1^] calculated from nanoITC data range from 10^3^ to 10^9^ [L·mol^-1^], reflecting both the concentration of macromolecules and the binding affinity. These values are generally classified as low (10^3^–10^4^ [L·mol^-1^]), moderate (10^4^–10^8^ [L·mol^-1^]), or high affinity (10^8^–10^9^ [L·mol^-1^]) (Velázquez Campoy and Freire, 2005). Both for Salt4-HSA and Salt4-AGP complexes, the obtained K_a_ [L·mol^-1^] values were in the order of magnitude 10^6^ [L·mol^-1^] (Table 9). The differences in K_a_ [L·mol^-1^] obtained using nanoITC (on the order of 10^6^) and fluorescence spectroscopy (on the order of 10^4^) result from the different physicochemical principles underlying these techniques. The nanoITC method determines the binding constant based on the general thermal effect of complex formation, regardless of the binding site, whereas fluorescence spectroscopy monitors changes in the local environment of specific fluorophores (tyrosine and tryptophan residues). As a result, association constants determined by the nanoITC method may be one to two orders of magnitude higher than those obtained by fluorescence spectroscopy. Nevertheless, the K_a_ values obtained for both complexes are within the range of moderate affinity (Ràfols et al., 2014). Furthermore, it can be observed that Salt4 binds to HSA and AGP with the reaction stoichiometry (n) of 3.71 ± 0.01 and 2.38 ± 0.16, respectively. Bou-Abdallah et al. (2016) demonstrated that binding stoichiometry provides crucial information about the interaction between ligands and proteins. For naproxen-HSA, diclofenac-HSA, and naproxen-BSA complexes, n values oscillated between 2.82 ± 0.32 and 3.31 ± 0.38. The authors explained this phenomenon that three ligand molecules bind to one protein molecule or to three distinct binding sites. Moreover, when stoichiometry is close to unity, it correlates with stronger binding affinity and more favorable enthalpy contribution, which highlights the relationship between binding site occupancy and interaction strength (Velázquez Campo and Freire, 2005; Bou-Abdallah et al., 2016). Based on the Bou-Abdallah et al. (2016) theory, it can be assumed that probably approximately four Salt4 molecules bind to one HSA molecule, and approximately two Salt4 molecules bind to one AGP molecule. Comparing the data collected in Tables 6, 7 with the data obtained using nanoITC (Table 9), it can be supposed that approximately four Salt4 molecules can bind to HSA molecules in one class of high-affinity binding sites, probably in subdomain IIIA, while approximately two Salt4 molecules may bind to one AGP clinically relevant binding site (QR binding site).

nanoITC provides detailed thermodynamic information about ligand-protein interaction. While the ΔG [kcal·mol^-1^] reflects the overall spontaneity and favorability of binding, the corresponding values of ΔH [kcal·mol^-1^] and ΔS [cal·mol^-1^·K^−1^]) reveal the dominant forces stabilizing the complex. It is worth noting that interaction with comparable ΔG but different ΔH and ΔS values indicates the presence of different intermolecular interaction, reflecting the varied nature of binding mechanisms (Gibbs, 1873). The binding of Salt4 to HSA and AGP molecules is characterized by high positive values of ΔH and ΔS, as well as negative values of ΔG (Table 9). This may indicate that the binding of Salt4 to model carrier proteins occurs spontaneously (Owczarzy et al., 2025a), and both Salt4-HSA and Salt4-AGP complexes are predominantly stabilized by hydrophobic forces or hydrophobic interactions, which is consistent with the classification proposed by Ross and Subramanian (1981). However, recent studies indicate that thermodynamic parameters similar to those obtained in the present study (ΔH>100 and ΔS>300) may often reflect molecular phenomena that are more complex than hydrophobic interactions. In particular, this refers to the processes of desolvation and solvent reorganization associated with the movement of water molecules located at binding sites within the protein molecule and surrounding the ligand during the formation of ligand-protein complexes. This is a common cause of a significant increase in the obtained ΔS values (Ladbury, 1996; Maurer and Oostenbrink, 2019). The high entropy values observed in this study suggest that the desolvation process may also be a key component of the binding mechanism of the studied complexes. This explanation is consistent with the experimental identification of hydrophobic interactions as a result of high positive values for both ΔH and ΔS (Owczarzy et al., 2025b; Ràfols et al., 2014; Ross and Subramanian, 1981), particularly when numerous water molecules are displaced from relatively hydrophobic regions of the binding site (Maurer and Oostenbrink, 2019). Importantly, the entropy changes observed for Salt4–HSA and Salt4–AGP exceed the values commonly reported for many protein–ligand systems, suggesting that complex formation is accompanied by significant restructuring of the hydration layer. Consequently, although the obtained thermodynamic parameters indicate a significant contribution from hydrophobic interactions, the high positive values of both ΔH and ΔS recommend that desolvation and solvent reorganization can play an important role in complex formation. Furthermore, the possibility of binding-induced conformational rearrangements cannot be excluded. Consequently, the interaction of Salt4 with HSA and AGP should be interpreted as a multifactorial process involving hydrophobic effects, dehydration of the binding site, solvent reorganization, and structural adaptation of the protein (Ladbury, 1996; Maurer and Oostenbrink, 2019).

## 5 Conclusion

The aim of this study was to analyze the interaction between Salt4 with anticancer potential and the main carrier proteins, such as HSA and AGP using spectroscopic (spectrofluorimetry, UV-Vis spectroscopy, circular dichroism spectroscopy) and calorimetric (isothermal titration nanocalorimetry) techniques. Based on the conducted studies, it was established that Salt4 can form predominantly static and stable complexes with HSA and AGP with moderate affinity, but more stable with HSA than with AGP. In addition, Salt4 increases the hydrophilicity of the environment around the aromatic amino acid residues present in the studied macromolecules, thus it may affect their tertiary structure. Based on far UV circular dichroism spectra and the calculated individual secondary structures content (α helix and β sheet) of the studied macromolecules in the absence and presence of Salt4, it can be concluded that Salt4 might not cause conformational changes in the secondary structures of HSA and AGP and therefore probably does not alter their biological functions in the human body. Analysis of the obtained data using both spectroscopic and nanocalorimetric techniques allowed us to conclude that approximately four Salt4 molecules bind to one HSA molecule in one class of high-affinity binding sites preferentially in subdomain IIIA, and approximately two Salt4 molecules can bind to one AGP molecule in a binding site with clinical significance. The calculated thermodynamic parameters indicate that the formation of Salt4 complexes with HSA and AGP is a spontaneous, multifactorial process involving hydrophobic interactions, dehydration of the binding site, solvent reorganization, and structural adaptation of the protein; hydrophobic interactions may be the dominant type of bond responsible for the stability of the studied complexes. Depending on the dose, Salt4 may cause both therapeutic and toxic effects. In addition, Salt4 can exhibit a promising profile of physicochemical, pharmacokinetic, and toxicological properties, consistent with the requirements for drug candidates. Although the results obtained are preliminary, they provide a significant contribution to the advancement of pharmaceutical science and the development of new drugs. Nevertheless, it is important to continue and expand research to include other blood serum carrier proteins, deoxyribonucleic acid (DNA) as the main molecular target of cancer therapy, as well as biological models and molecular targets to confirm the Salt4 potential pharmacological significance.

## Data Availability

The original contributions presented in the study are included in the article/supplementary material, further inquiries can be directed to the corresponding author.
